# Molecular Mechanisms Underlying Sensory and Chemical Changes in Muscle Foods Induced by Sous-Vide Cooking: A Review

**DOI:** 10.3390/foods14172967

**Published:** 2025-08-26

**Authors:** Qingqing Jiang, Ruiying Lv, Panpan Zhai, Xichang Wang, Yuan Li, Mingyu Yin

**Affiliations:** 1 College of Food Science and Technology, Shanghai Engineering Research Center of Aquatic Product Processing & Preservation, Shanghai Ocean University, Shanghai 201306, China; qqjiang@shou.edu.cn (Q.J.); 15837028985@163.com (R.L.); xcwang@shou.edu.cn (X.W.); 2 Department of Microbiology, ADA Forsyth Institute, Somerville, MA 02143, USA

**Keywords:** sous-vide cooking, sensory attributes, protein changes, lipid changes, muscle foods

## Abstract

Sous-vide cooking has attracted considerable attention for its capacity to minimise nutritional and qualitative degradation in muscle foods, with research into the intricate changes in sensory attributes becoming a particular focal point. This paper presents recent insights into the sensory transformations of meat, especially shifts in texture, color, and flavour during sous-vide processing. Both cooking temperature and duration exert important influences, with temperature generally playing a more pivotal role across diverse conditions. The molecular mechanisms underlying the impacts of sous-vide cooking on muscle foods are comprehensively delineated. Notably, partial protein denaturation is a critical factor influencing textural changes. Compared with conventional thermal cooking, certain endogenous enzyme activities are retained during sous-vide processing. Furthermore, alterations in protein structure affecting water migration are crucial for meat juiciness. Flavour development is closely linked to lipid oxidation and its interplay with amino acids. To advance understanding in this domain, the application of mass spectrometry, omics, and other advanced analytical techniques is deemed imperative. This review provides comprehensive insights into the sensory and molecular changes occurring in meat during sous-vide cooking, which offer valuable guidance for researchers and industries to understand the underlying mechanisms and optimise culinary techniques in this domain.

## 1. Introduction

Meat and meat products, rich in nutrients like protein and lipids, are a crucial component of the diet for numerous people worldwide. They provide the body with ample energy and nutrition. According to data from the Food and Agriculture Organization (2023) [1], approximately 346 million tons of meat have been produced and processed globally each year since 2020. It is predicted that global per capita meat consumption will increase at an annual rate of 0.1%. The pursuit of minimising nutrient loss and preserving food texture has driven scientific exploration in thermal cooking methods. Traditional methods of cooking meat, such as steaming, frying, pan-frying, and grilling, are typically carried out at high temperatures. High temperatures can induce alterations in the internal tissue structure of meat and a decline in tenderness, thus increasing its toughness. Moreover, these changes are accompanied by protein denaturation, lipid oxidation, degradation of heat-sensitive vitamins, and decomposition of bioactive compounds. In addition, this process leads to a reduction in beneficial fatty acids and promotes the formation of harmful oxidative products. Generally, lower temperature/time combinations aim to retain the original state of food, considering the positive correlation between molecular activity and temperature, where the cumulative chemical reactions are integral over the time of such activity [2]. However, short-time treatments at low temperatures may fail to adequately eliminate foodborne microorganisms, compromising the fundamental principle of food safety [3]. Attempts to strike a balance between safety and sensory aspects involve implementing non-thermal sterilization techniques and integrating thermal processes with other physical methods to optimize food quality. Sous-vide cooking stands out among these physical methods.

Derived from the French words “sous” (under) and “vide” (vacuum), sous-vide cooking originated in 1970s France. It refers to the method of preparing raw ingredients by cooking them within heat-stable, food-grade vacuum-sealed packaging under precisely controlled temperature and time conditions [4]. This method represents a meticulous low-temperature cooking technique. It entails heating food that has been vacuum-sealed within a water bath, sustaining it at a precisely regulated temperature over an extended duration. The core of this technology is to seal ingredients in vacuum bags and cook them at low temperatures for long periods of time, adjusting the temperature and time parameters to suit different ingredients. For example, braised pork cooked under vacuum at a low temperature of 70 °C for 8 h or 75 °C for 8 h is even better [5]. Goose meat is more effective at 60 °C for 4 h [6]. Initially embraced by high-end restaurants, sous-vide cooking was notably linked to W.R. Grace—an American packaging firm that held a patent encompassing the core features of this culinary technique [7]. Initially centered on minimizing nutrient loss and drip loss through optimized cooking conditions, this method garnered attention from the U.S. Food and Drug Administration (FDA) in 1988, amid concerns about pathogenic bacterial growth in sous-vide products that were insufficiently heated. Meanwhile, taste tests conducted on sous-vide products revealed heightened flavors in lamb and improved sensory experiences with duck from Home Rouxl, a UK-based producer [7]. Subsequently, in the early 21st century, the parameters of sous-vide cooking came under scrutiny for their effects on food quality and sterilization efficiency.

Since its inception in the 1970s by a French chef, sous-vide cooking has gained popularity in prestigious restaurants. Its appeal lies in the convenience of managing prepared ingredients while preserving maximum nutritional value. In contrast to traditional culinary practices, chefs employing this method typically use lower temperatures and longer cooking times [8]. For instance, in the culinary industry, pork is typically processed at temperatures between 75 and 80 °C, whereas sous-vide chefs use temperatures ranging from 60 to 63 °C for the same meat [9]. The widespread availability of vacuum-sealing machines and thermostatted water baths has facilitated the global adoption of sous-vide cooking in restaurants. Published recipes have further improved their accessibility. With the rising popularity of molecular gastronomy and growing demand for higher food quality, the sous-vide method is expected to gain increasing attention in everyday cooking.

In the realm of sous-vide processing, the minimized loss of nutrients and sensory attributes is attributed to meticulous temperature control and the protective barrier of vacuum packaging. Precise temperature control is pivotal in sous-vide cooking for optimizing food quality, while processing time affects microbial residues. Table 1 presents parameters from recent research, showing that sous-vide cooking temperatures for meat or fish typically range from 50 °C to 85 °C, with cooking times varying from 9.48 min to 72 h depending on the food’s type and size. Unlike non-thermal food processing methods, sous-vide achieves microbial inactivation primarily through precise temperature regulation, which mitigates potential genetic risks associated with mutations and enhances safety measures [10]. Furthermore, sous-vide requires relatively simple, cost-effective equipment and operational environments, with vacuum packaging providing an additional safeguard against external contaminants [11]. These attributes collectively establish sous-vide as a preferred method for producing high-quality foods. However, several crucial considerations warrant special attention. Achieving precise temperature control is paramount in sous-vide equipment, as temperature-related reactions are irreversible [12]. Notably, protein degradation and moisture migration are highly temperature-dependent, both of which significantly influence the texture of meat and seafood. Safety, a primary concern in food processing, necessitates a balance between maintaining sensory quality and ensuring bactericidal efficacy in sous-vide cooking. This is particularly relevant given the risk of microbial residues associated with low-temperature cooking [13]. Furthermore, specific reactions that impart unique flavors or affect color in food processing, such as the Maillard reaction, require temperatures between 140 °C and 170 °C [14]. This highlights the challenge of preserving a food’s inherent quality and natural reactions at lower temperatures, rather than inducing complex new sensory characteristics.

Given the inherent complexity of food systems, variations in type, shape, and arrangement can yield diverse outcomes even under standardized sous-vide conditions. Muscle foods, particularly meat and seafood, exhibit distinct differences in protein composition, water content, and microbial growth patterns, which complicates parameter selection for sous-vide processing. Exploring the preservation mechanisms of sous-vide cooking clarifies the path toward informed parameter choices in food processing. This work reviews recent advancements in sous-vide cooking as applied to muscle foods, delineating the mechanisms that preserve nutritional and sensory properties while outlining potential directions for future research.

## 2. Sensory Changes During Sous-Vide Cooking

Beyond mere sterilization, achieving optimal sensory qualities in cooked meat is a key goal of thermal cooking. Sensory evaluation primarily focuses on flavor, color, and texture. Proper thermal processing plays a critical role in reducing the unpleasant odors of raw meat, imparting it with pleasant aromas, and tenderizing its texture to enhance palatability [45]. However, excessive heat exposure can significantly affect the sensory properties of meat, triggering a host of complex reactions when subjected to prolonged time and elevated temperatures. Precisely regulating reactions at specific temperatures, such as the Maillard reaction and caramelization, can impart distinctive flavors or colors to food [14]. Yet, for a multifaceted food system like meat products, it is erroneous to assert an absolute rule that either higher or lower temperatures are more suitable for optimizing sensory attributes, as sensory preferences may vary across different target populations or dietary backgrounds.

Unlike traditional thermal methods, sous-vide cooking preserves texture and original flavors more effectively due to milder chemical reactions and reduced moisture loss. However, the changes induced by sous-vide cooking cannot be overlooked when compared to the characteristics of raw meat. This section seeks to examine and clarify the sensory alterations observed in muscle foods during sous-vide cooking.

### 2.1. Flavour Changes During Sous-Vide Cooking

Flavor is a critical determinant of food quality, influencing consumption through taste and smell interactions. These sensory experiences are primarily determined by volatile compounds, amino acids, lipids, nucleotides, and so on [46]. Thermal cooking significantly alters the flavor profile of muscle foods, which reduces the blood-like taste of raw materials and triggers the generation of new aromas through reactions such as lipid oxidation, vitamin degradation, and protein denaturation [47], thereby contributing to the formation of various flavour-active molecules.

Flavor changes in meat processing primarily stem from lipid degradation and oxidation, the Maillard reaction, and interactions between Maillard reaction products and lipid degradation products [48]. Both temperature and duration of heat exposure significantly influence the production of volatile compounds. Maillard reactions intensify notably above 140 °C, while lipid oxidation and degradation are temperature-sensitive processes. Consequently, the mild processing parameters of sous-vide cooking reduce the generation of certain volatile compounds compared to high-temperature treatments.

Key volatile components in sous-vide cooked meat include aliphatic and aromatic hydrocarbons, alcohols, ketones, aldehydes, sulfur compounds, and esters. Typically, the types of these compounds remain consistent before and after sous-vide cooking. However, their individual concentrations fluctuate during processing, which imparts distinct flavors to the products. In the study of Yuan et al. [15], the primary flavour compounds identified in tuna meat under different processing conditions were aldehydes and alcohols, followed by alkanes, ketones, and esters. Nevertheless, with the increase in processing temperature, the relative concentrations of most of these compounds exhibited an upward trend. In sous-vide cooking of duck meat under different temperature-time combinations, among all volatile components, including aldehydes, alcohols, ketones, and esters, aldehydes account for the highest proportion. Specifically, as cooking time increases, the total concentration of aldehydes first increases and then stabilizes at 60 °C, while it initially rises before decreasing at 70 °C; and it shows a decreasing trend at 80 °C [49]. Different compounds exert varying effects under diverse time-temperature combinations, thereby influencing flavor outcomes. Previous studies have highlighted that sous-vide cooking intensifies the flavor of poultry meat [50]. Other investigations have found that sous-vide-cooked largemouth bass, braised pork, and Nile tilapia exhibit a more enhanced aroma compared to their traditionally cooked counterparts [5,51,52].

Cooking under higher temperatures results in extensive lipid oxidation reactions and complex interactions between amino acids and other compounds. The Maillard reaction is critical to meat flavor development, which is significantly affected by sous-vide temperatures around 80 °C. Particularly, the process of Strecker degradation is influenced by sous-vide parameters, thereby affecting volatile compounds derived from amino acids and specific branched aldehydes [53]. Temperature plays a more significant role than time in the formation of these compounds, with different compounds exhibiting varying sensitivities [54]. Because Strecker degradation generally contributes positively to flavor development, certain substances such as dimethyl disulfide, 1-hydroxy-2-propanone, and 2-methyl thiophene may not form at sous-vide cooking temperatures.

The time and temperature of sous-vide cooking influence the changes in aliphatic hydrocarbons. It has been found that the contents of 2,3,4-trimethylpentane, pentane, 1-hexene, methylcyclopentane, 2-methylpentane, 2-methylheptane, 3-methylhexane, heptane, methylcyclohexane, 1,2,3-trimethylcyclopentane, and decane are higher in meat cooked at elevated temperatures [55]. During thermal processing, hydroperoxides generate various secondary derivatives, which contribute to the formation of volatile compounds such as hydrocarbons.

Sugar degradation via Maillard reaction produces hydroxiketones, which are detected in traditionally thermally cooked meats, and go on to form pyrazines, oxazolines, oxazoles, and thiazoles [56]. Due to its relatively mild temperatures, sous-vide processing does not induce the formation of these compounds, thus preserving the original flavour profile.

Furan formation occurs through multiple pathways, including high-temperature induction and oxidation of unsaturated fatty acids. It can also arise from naturally occurring precursors in foods, such as carbohydrates, amino acids, ascorbic acid, fatty acids, and carotenoids. They are primarily produced via Maillard reactions and oxidation processes, which typically require higher temperatures [57]. Under sous-vide conditions, furan is primarily derived from lipid oxidation rather than precursor-based formation. Additionally, sous-vide cooking increases thiobarbituric acid reactive substances (TBARS) and certain lipid oxidation-derived volatiles (*p* < 0.001), but it concurrently reduces aroma compounds in cooked meat [58].

### 2.2. Colour Changes During Sous-Vide Cooking

Sous-vide cooking often imparts a lighter appearance to meat compared to conventional methods due to surface water exudation, which is influenced by cooking temperatures. Lower sous-vide temperatures result in reduced lightness, while higher temperatures induce significant protein aggregation, bringing greater brightness on the meat surface. Conversely, darker meat surfaces occur when higher cooking temperatures increase moisture penetration, intensifying the denaturation and aggregation of myofibrillar and sarcoplasmic proteins [59,60].

The redness of cooked meat is closely associated with the denaturation and concentration of myoglobin. Myoglobin undergoes denaturation within the temperature range of 55 °C to 65 °C, and this process becomes more pronounced as temperatures rise to 75 °C or 80 °C, which in turn has an inverse effect on meat redness. In comparison to meat cooked in boiling water, sous-vide-cooked meat displays greater redness, especially at lower temperatures. This phenomenon reflects differences in myoglobin degradation patterns that are associated with varying cooking temperatures [61,62,63].

The characteristic brown colouration observed in cooked meat at high temperatures is less prominent in sous-vide-cooked meat, which is attributed to the occurrence of the Maillard reaction. At elevated temperatures, the precipitation of meat juices rich in flavour precursors actively contributes to the Maillard reaction, via which coloured compounds are generated. Additionally, the formation of metmyoglobin and heat-induced denaturation processes still contribute to the brownish hue observed in sous-vide-cooked meat [64].

The colour of fresh meat is contingent on the content and physicochemical state of myoglobin. It is manifested in different hues, namely purple (reduced myoglobin), red (oxymyoglobin), and brown (metmyoglobin). Ribs cooked sous vide at lower temperatures and for shorter durations exhibit higher lightness, attributable to the increased free water content on the surface. The use of laminate pouches reduces the oxidation of red myoglobin to brown metmyoglobin; consequently, the significant impacts of sous-vide temperature and duration on the hue angle and chroma defined by the International Commission on Illumination (CIE) are minimized [65]. The impact of temperature on colour changes in sous-vide-cooked meat is significantly more pronounced than that of cooking duration. The denaturation of myoglobin at temperatures exceeding 70 °C results in distinct colour alterations. However, certain challenges arise in poultry, particularly chicken breasts, due to their tendency to retain a pinkish hue. It is a characteristic that may mislead consumers into perceiving the meat as undercooked or bloody, thereby causing dissatisfaction. This consumer perception has limited the extent of research on sous-vide-cooked chicken breasts relative to other sous-vide-prepared foods. Additionally, the selection of packaging materials exerts a significant influence on meat quality and shelf life, particularly with regard to oxygen permeability and its subsequent effects on colour parameters [50].

### 2.3. Texture Changes During Sous-Vide Cooking

The sensory experience of meat is strongly dependent on its texture, encompassing tenderness, chewiness, cohesiveness, hardness, springiness, resilience, and adhesiveness. Tenderness, an attribute influenced by cooking temperatures, tends to be superior at lower temperatures, as exemplified by sous-vide cooked beef roasts prepared at 60 °C compared to those cooked using traditional methods [65]. Shear force, which is inversely related to tenderness, correlates positively with moisture content, and lower shear force values indicate more desirable meat texture [66].

Sous-vide cooking within specific temperature ranges can impact meat toughness. For instance, tenderness decreases at temperatures of 40–50 °C and 60–80 °C but increases between 50 and 60 °C, possibly due to collagen shrinkage and denaturation of perimysial connective tissue [67]. The connective tissue strength, reflected in adhesion, diminishes at temperatures of 50–60 °C, correlating with decreased perimysial connective tissue strength [68]. Sous-vide-cooked meats, like pork fillet and chicken breast, exhibit lower springiness, chewiness, hardness, and gumminess compared to meats cooked in 200 °C ovens [69]. Reduced hardness is observed in sous-vide-treated pork, with slight differences in cohesiveness and springiness [35].

Collagen solubilization has an inverse relationship with the rarity, juiciness, and shear force of beef, which increases with prolonged cooking [70]. Conversely, chewiness tends to decrease at lower temperatures. However, cooking at 75 °C may induce heat-related toughening, potentially linked to crust formation and resulting in a chewier meat texture [71].

### 2.4. Consumer Taste Changes During Sous-Vide Cooking

Cooking methods can alter the sensory properties and consumer experience of food by regulating heat conduction and chemical reaction pathways. Dry-heat methods such as grilling and frying promote the Maillard reaction and lipid oxidation, generating heterocyclic compounds (e.g., pyrazines, thiazoles, and furans) and carbonyl volatiles, which impart a “smoky-charred” profile and complex flavor layers to meat. Simultaneously, rapid evaporation of surface moisture forms a crispy crust while locking in internal juices, further enhancing flavor. Wet-heat methods, including steaming, boiling, and sous-vide cooking, better preserve umami amino acids. In terms of texture, high-temperature short-time processing renders the surface crispy and accentuates internal juiciness, whereas low-temperature long-time processing induces collagen hydrolysis, improving tenderness and chewability, which is a characteristic particularly favored by older consumers. The research of Li et al. [72] demonstrated that sous-vide cooked chicken breast samples exhibited lower cooking loss and shear force compared to traditionally boiled samples.

Traditional sensory evaluation involves first training and selecting assessors, followed by determining the scoring scales and weights for the samples to be evaluated in accordance with national standards for sensory assessment. Currently, sensory evaluation often integrates traditional methods with mathematical approaches or instrumental analysis. Mathematical methods are employed to quantify sensory assessments; alternatively, after sensory scoring, instruments such as texture analyzers or electronic noses are used to analyze sample components, establishing a correlation model between instrumental analysis data and sensory scores. This enables instrumental analysis data to reflect sensory evaluation results. In the study of Ding et al. [73], the human sensory evaluation results indicated that Buddha’s hand fruit is primarily sour and sweet, with a soft texture and subtle flavours of saltiness, grapefruit peel, licorice, tea, hawthorn, fragrance, and cinnamon. These findings were confirmed by E-tongue analysis, which confirmed that sweetness and sourness are the primary flavours.

## 3. Changes in Muscle Composition During Sous-Vide Cooking

Macroscopic sensory changes arise from the superimposition of reactions and interactions among different food components at the molecular level during the sous-vide cooking process. Despite variations in specific ingredient ratios and properties, meats share the same major components, namely water, proteins, lipids, and free amino acids. The change patterns of different meat components during sous-vide cooking, the existing mechanisms, and hypotheses are summarized in this section.

### 3.1. Lipid Changes During Sous-Vide Cooking

Lipid oxidation is a complex process in which hydroperoxides and other primary oxidation products are generated from polyunsaturated fatty acids. Following primary autoxidation, a series of secondary reactions occur, leading to the degradation of hydroperoxides and the formation of a broad range of compounds. For instance, volatile aldehydes such as hexanal and 2,4-decadienal are produced through the oxidation of linoleic acid, and these compounds impart off-flavours to oxidized meat and meat products, which have also been widely used as indicators of lipid oxidation. Both cooking time and temperature exert considerable effects on lipid oxidation in muscle foods by inducing radical formation, which can ultimately impact the nutritional value and sensory properties of meat [74]. Generally, heat can promote lipid oxidation, and increasing temperature accelerates the rate of lipid oxidation [75,76]. It has been observed that temperature affects the fatty acid content in sous-vide-cooked sheep meat, and processing at lower temperatures results in higher fatty acid contents. At higher cooking temperatures, the secondary compounds resulting from lipid oxidation increase, and a small number of carbonyls from lipid oxidation bind to proteins [77]. Cooking methods can also influence lipid oxidation levels. For sous-vide cooked pork, higher temperatures and longer cooking times lead to greater lipid oxidation [63].

During the early stage of lipid oxidation, conjugated dienes are formed through the rearrangement of double bonds in hydroperoxides derived from polyunsaturated fatty acids [63]. During the heating of meat, the content of conjugated dienes increases as lipid oxidation proceeds [63]. The thiobarbituric acid reactive substances (TBARS) value is an indicator reflecting the content of secondary lipid oxidation products. Thermal processing leads to a higher TBARS value compared to raw meat [78]. However, during sous-vide cooking, the TBARS value decreases because these reactive substances can further react with compounds containing primary amino groups (such as DNA, proteins, phospholipids, and amino acids). Pork cheeks cooked at 80 °C exhibit a lower TBARS value than those cooked at 60 °C [63].

It has been reported that lipid oxidation significantly influences the flavour of sous-vide-cooked meat products. Cooking at higher temperatures can promote the generation of volatile compounds from lipid oxidation, including butanal, nonanal, 2-octanone, and certain furanones and furans [79]. Higher temperature treatments can also positively facilitate the degradation of unsaturated fatty acids. The generated free radicals further drive the decomposition of other fatty acids, forming volatile compounds such as octanal, nonanal, and heptanal, which impart unique flavours to meat. At lower sous-vide temperatures, the extent of flavour changes is less pronounced due to reduced lipid oxidation. During sous-vide cooking, the accumulation of lipid oxidation products shows little increase with prolonged cooking time.

Aldehydes are key products of lipid oxidation during the thermal processing of meat, with hexanal being particularly notable [80]. Straight-chain aliphatic hydrocarbons are also essential compounds derived from lipid oxidation and are primarily identified in sous-vide-cooked meat. Thermal processing can accelerate lipid oxidation, especially the oxidation of polyunsaturated fatty acids, thereby increasing the number of free radicals. The generated free radicals can attack other less oxidation-susceptible fatty acids, such as oleic acid. As the temperature and duration of sous-vide cooking increase, the content of most aldehydes decreases. At higher temperatures, aldehydes react with the amine groups of lysine, cysteine, and glutathione to form other volatile substances [81]. The decrease in their concentrations under higher temperatures and longer cooking times likely reflects their involvement in further reactions post-formation, generating other volatile and non-volatile products [81]. Roldan et al. [77] found that the content of 3-methylbutyraldehyde increased significantly with cooking time during sous-vide processing at 80 °C and rose nearly sixfold from 6 to 24 h. As a marker for the progression of amino acid degradation via the Strecker reaction, such changes were far less pronounced at lower temperatures. As cooking time prolonged in the sous-vide cooking of lamb loins at 80 °C, the content of secondary lipid oxidation compounds decreased, while the number of lipid oxidation-derived carbonyls bound to proteins remained low. In these samples, the content of 3-methylbutanal increased, which was formed by the attack of lipid-derived carbonyls on leucine [77]. Carbonyls, α-aminoadipic semialdehyde, and γ-glutamic semialdehyde are potential aldehyde moieties that can react with the amino groups of amino acids to form Schiff bases, which is the initial step in the Strecker degradation of amino acids [82].

It has been reported that high temperatures can stimulate lipid peroxidation [83]. In contrast, sous-vide cooking, particularly at lower temperatures, can limit the extent of lipid peroxidation, thereby reducing the content of hydrocarbon compounds. Hydroperoxides and free radicals generated from lipid oxidation can react with susceptible amino acids, while some lipid carbonyls can interact with amino acids to induce Strecker degradation, producing volatile compounds such as Strecker aldehydes and certain Maillard-like volatile compounds. Table 2 shows the effects of vacuum low-temperature cooking on lipid oxidation in meat.

### 3.2. Protein Changes During Sous-Vide Cooking

Proteins are vital components of meat. During sous-vide cooking, changes in meat texture are closely linked to protein denaturation and degradation. Analyzing the extent of denaturation and functional alterations of different proteins throughout the process is crucial for understanding how meat quality changes during sous-vide cooking.

#### 3.2.1. Effects of Protein Changes on Meat Texture

Total volatile basic nitrogen (TVB-N) is a key parameter reflecting the degree of protein denaturation. Meat cooked using sous-vide methods exhibits a lower TVB-N value compared to traditionally cooked meat. Moreover, as cooking time and temperature increase, the TVB-N value decreases, indicating that sous-vide cooking can retard protein denaturation in a time- and temperature-dependent manner. Since TVB-N is primarily produced through protein degradation, an elevated TVB-N value typically signifies texture deterioration. The research of Palamae et al. [87] demonstrated that samples cooked via sous-vide cooking exhibited a slower increase in TVB-N content, highlighting the effectiveness of this cooking technique in extending the shelf life of seafood. In the study of Mohan et al. [88], sous-vide cooking of Indian white shrimp significantly inhibited the formation of TVB-N, indole, and lipid oxidation, thereby extending the product’s shelf life.

The collagen solubilisation, protein denaturation and fibre shrinkage are heat-induced changes. While collagen solubilisation can have a tenderising effect on meat texture, the other two changes linked to myofibrillar proteins may increase toughening [89]. Under sous-vide cooking conditions, although myofibrillar-based toughening is unintended, intense collagen solubilisation occurs, resulting in gelatin formation [63]. Generally, collagen shrinks within the range of 62–68 °C. The partial denaturation and shrinkage of collagen fibres in connective tissue between 50 °C and 60 °C are possibly responsible for the increased tenderness of meat during cooking at low temperatures. Some previous studies attribute the increased meat tenderness to the unfolding of the collagen triple helix by a collagenase-like enzyme, allowing other proteolytic enzymes to degrade it. In contrast, other studies have reported varying results regarding the temperatures at which collagen is solubilised. Wu, Dutson, and Smith [90] indicate that significant collagen solubilisation does not occur until 80 °C. At lower temperatures (below 80 °C), meat toughness decreases, potentially due to the weakening of collagen.

The content of soluble collagen is negatively correlated with meat toughness. During the sous-vide cooking process, collagen may undergo enzymatic breakdown and heat-induced denaturation. Tornberg [91] indicates that within the range of 50–65 °C, meat fractures more easily due to the bundling of collagen fibres and the denaturation of sarcoplasmic proteins. The potential reasons for meat tenderisation can be summarised as both increased collagen solubilisation and the weakening of structure-related proteins. The enhanced tenderness of sous-vide-cooked meat may result from the weakening of myofibrils caused by the action of proteolytic enzymes and increased collagen solubilisation. Furthermore, the bioactivity of collagenase and other proteolytic enzymes is inactivated at higher temperatures, which can affect collagen solubilization. The bioactivity of these enzymes may remain below 60 °C, allowing certain reactions to continue. Research by Brüggemann, Brewer, Risbo, and Bagatolli [92] provides evidence for this phenomenon using second harmonic generation microscopy. Collagen shrinkage occurs at 57 °C, while the signal corresponding to collagen disappears at 59 °C.

Thermal processing can induce the denaturation of myofibrillar proteins and the solubilisation of connective tissues. The solubilisation of connective tissues results in meat tenderisation, while the denaturation of myofibrillar proteins can lead to meat toughening. These changes in proteins cause water loss from muscle tissue, resulting in meat toughening. Between 50 °C and 60 °C, the aggregation and denaturation of myofibrillar proteins can increase the degree of scattering, enhancing the clarity of cooking drip. At higher sous-vide cooking temperatures, myoglobin denatures more severely, leading to lower solubility and reduced redness in cooking drip. Differential scanning calorimetry results indicate that purified myoglobin begins to denature at approximately 60 °C and can precipitate with certain other proteins [61]. However, in the presence of other proteins, the denaturation temperature of myoglobin may decrease to some extent.

Another significant change between raw meat and heated meat is the transformation of a viscoelastic material into an elastic one. The texture of raw meat is relatively rigid due to viscous flow within the fluid-filled channels between myofibres and myofibre bundles. When heated to around 65 °C, sarcoplasmic proteins aggregate and form a gel, making the meat easier to chew. In the temperature range of 65–80 °C, the increased elastic modulus requires greater tensile stress to propagate fractures, resulting in a firmer texture in heated meat [91]. The partial denaturation of certain proteins under sous-vide cooking can be a key reason for meat’s fine texture. Some endogenous enzymes retain partial activity after processing. It has been found that the residual collagenolytic activity is detected in meat cooked at 60 °C for 6 h, and the collagenase activity remains measurable at 60 °C [91]. This can contribute to meat tenderness following prolonged cooking.

Texture changes in meat are the combined effects of protein complexes. In Baldwin’s study [91], meat cooked at 70 °C and 80 °C exhibited higher hardness values compared to that cooked at 60 °C, which might be attributed to the denaturation of myosin (55–60 °C), actin (≈80 °C), and the contraction of collagen (56–65 °C). However, the study by Roldán et al. [77] did not show an equivalent difference in hardness for the samples cooked at the same temperature over a longer period. In these latter samples, collagen might have sufficient time to solubilise completely, counteracting the hardness caused by myofibrillar protein shrinkage and resulting in reduced hardness. As heating time increased, the salt-soluble protein content decreased significantly. Between 50 °C and 65 °C, the contraction of collagen fibres reduces the breaking strength of connective tissue, while myofibrillar proteins (the main salt-soluble proteins in muscle) undergo only minor changes and begin to form weak gels. At higher temperatures, myofibrillar proteins form a regular, dense network gel structure due to increased intermolecular disulfide bonds and enhanced hydrophobic interactions, leading to a reduction in crude protein content. Meat proteins are denatured by heat to form a network structure. The longer the heating time, the poorer the retention of this protein gel. When meat is heated at 75 °C, connective tissues denature and myofibres contract noticeably, forming a protein gel network with distinct gaps [5].

The most important textural effect of heat treatment is the tenderisation of meat, which occurs through the transformation (thermohydrolysis) of collagen at temperatures between 60 and 70 °C. Temperature is the primary factor in reducing collagen-induced hardness. Since poultry meat contains procollagen, an immature form of collagen that swells easily in aqueous environments, it does not require prolonged or intensive thermal treatment. The denaturation temperature of collagen has been reported to range from 53 °C to 63 °C, while myofibrillar proteins (primarily myosin) denature at 40–60 °C, followed by the gelation of collagen fibres at 60–70 °C and the denaturation of actin at 70–80 °C. Above 60 °C, muscle proteins denature, and myofibres undergo significant shrinkage in both diameter and length. The tenderisation achieved through sous-vide cooking is mainly attributed to reduced protein denaturation at the typically lower temperatures used, the weakening of connective tissue via collagen solubilisation, and water retention [50]. Prolonged heating in a humid in-pack environment, along with the overpressure created by saturated steam, further enhances collagen solubilisation and myofibrillar fragmentation [65].

#### 3.2.2. Protein Oxidation and Its Effects on Meat Quality

During thermal processing, cellular compartmentalisation is disrupted, releasing free catalytic iron. The cleavage of hydroperoxides leads to increased protein carbonylation, which is an indicator of the degree of protein oxidation [82]. Oxygen radicals generated during thermal processing can directly attack meat proteins or react with secondary oxidative stress products, including oxidised lipids and other pro-oxidants [74]. Protein polymerisation leads to reduced solubility, diminished enzyme activity, and decreased protein digestibility. Protein oxidation can affect the bioactivity and functionality of proteins, including the decrease in water-holding capacity, texture damage, and impaired protein digestibility. The formation of protein carbonyls can alter the overall electrical configuration of muscle proteins, compromising their functionality, reducing water retention, and damaging textural properties. The content of protein carbonyls, α-aminoadipic semialdehyde, and γ-glutamic semialdehyde is an indicator of the degree of protein oxidation.

There is a significant interaction in protein carbonyl content under different combinations of cooking temperature and time for lamb loin during sous-vide processing. However, the pattern of protein carbonyl accumulation over processing time is very similar across different cooking temperatures. The levels of α-aminoadipic semialdehyde and γ-glutamic semialdehyde increased with cooking time in samples cooked at 60 °C, whereas no such increase was observed in those cooked at 70 °C and 80 °C [77]. In the study by Roldán et al. [77], as sous-vide cooking time was extended, the cooking loss of lamb loins increased slightly alongside higher levels of α-aminoadipic and γ-glutamic semialdehydes. This suggests that limiting protein oxidation and denaturation under milder conditions may help muscle proteins retain water. In samples cooked at 80 °C, the levels of α-aminoadipic and γ-glutamic semialdehydes did not increase, likely due to the Strecker reaction between these aldehyde moieties and amino acids. Promeyrat, Daudin, and Gatellier [93] observed that, within the range of 45–90 °C, the content of protein carbonyls in myofibrillar mimetic models increased slightly with rising temperature, indicating that lower temperatures could help reduce the degree of protein oxidation to some extent. Over a relatively short period (300 s), sous-vide cooking time had little impact on protein carbonyl content [94], whereas over a longer duration (24 h), the degree of protein oxidation was positively correlated with extended processing time [77]. Heat can promote the generation of these two protein carbonyls [95], and these compounds can further degrade through other reactions [82]. The carbonyl groups in α-aminoadipic semialdehyde and γ-glutamic semialdehyde can induce a Strecker-type reaction with free amines [74]. The α-aminoadipic semialdehyde can react with non-modified amino acid residues, generating Schiff bases and cross-links [96]. Under severe oxidation conditions during cooking, the semialdehyde can be transformed into α-aminoadipic acid [95]. As temperature increases, the involvement of α-aminoadipic semialdehyde and γ-glutamic semialdehyde in the Strecker degradation of amino acids is enhanced. As cooking temperature increases and duration is extended, protein oxidation levels rise [97,98]. However, due to the particularly complex nature of the protein system in meat, proteins with different compositions and functions may exhibit varying sensitivity to cooking parameters.

**Figure 1:**
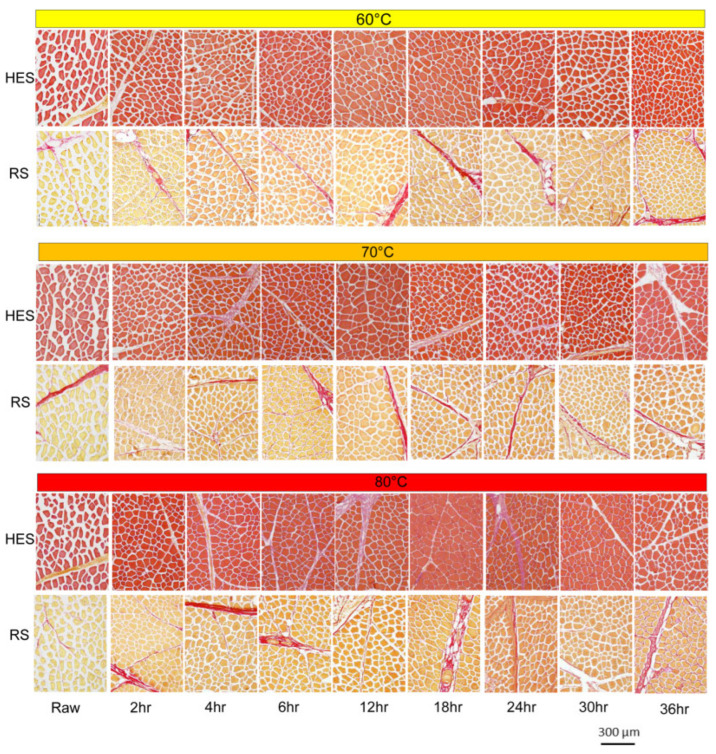
Effect of time/temperature interactions on muscle microstructure. Images acquired from sous-vide-cooked Thai beef (*Bos indicus*) semimembranosus muscle [99].

Figure 1 illustrates muscle structural changes as a function of temperature and heating time, using representative images of hematoxylin-eosin-saffron (HES) and Sirius red (SR) stained cross-sections. HES stains myofibres red and muscular connective tissue orange. SR stains intramuscular connective tissue red and myofibres yellow. The fibres remain clearly distinguishable regardless of cooking conditions. Although still identifiable, the connective tissue dissolves as cooking time increases. The shrinkage of myofibrillar mass during the first 2 h of cooking is a consequence of the increased heating rate, which is reflected by a decrease in the cross-sectional area of myofibrillar mass with rising temperatures. For samples cooked at 60 °C and 70 °C, the cross-sectional area of myofibrillar mass decreased progressively, with the 70 °C samples shrinking faster than those at 60 °C until 6 h of cooking. At 12 h, this area increased dramatically. The extramyofibrillar spaces remained stable until 6 h, then decreased sharply at 12 h of cooking, after which their time-course profile became more irregular. Myofibres heated to 80 °C exhibited less shrinkage compared to those heated to 60 °C and 70 °C. A larger lateral myofibre size at 80 °C than at 60–70 °C was also observed in muscle cooked at atmospheric pressure [97]. Therefore, this phenomenon appears to be independent of the sous-vide cooking method but linked to time- and temperature-dependent changes. The shrinkage of myofibres heated to 60 °C was driven by the thermal denaturation of collagen, while subsequent swelling at higher temperatures was attributed to an envelope of denatured collagen surrounding the myofibres, which enables the cells to retain their morphology despite water loss.

Actin denaturation is thought to shorten the thin filaments, leading to a rearrangement of denatured myosins [98] and likely altering the ultrastructure of the myofibrillar mass. Sarcoplasmic proteins are expected to precipitate and form a gel within the myofibres, which can modify their structure as the meat cools after cooking. Some of the proteins constituting the intermediate filaments also undergo denaturation within this temperature range. For example, titin has a denaturation temperature of around 76 °C. Purslow et al. [98] demonstrated that cytoskeletal proteins played little role in heat-driven shrinkage, but their samples were cooked using a different method than that employed here, so we cannot rule out a potential role for cytoskeletal protein denaturation in the myofibres’ shrinkage mechanism observed in this study. Thermal denaturation of proteins exposes hydrophobic groups, promoting hydrophobic interactions that form aggregates [100] and facilitate the gelling of myosin molecules and sarcoplasmic proteins. These sequential changes are likely to induce variations in the volume within myofibres.

**Figure 2:**
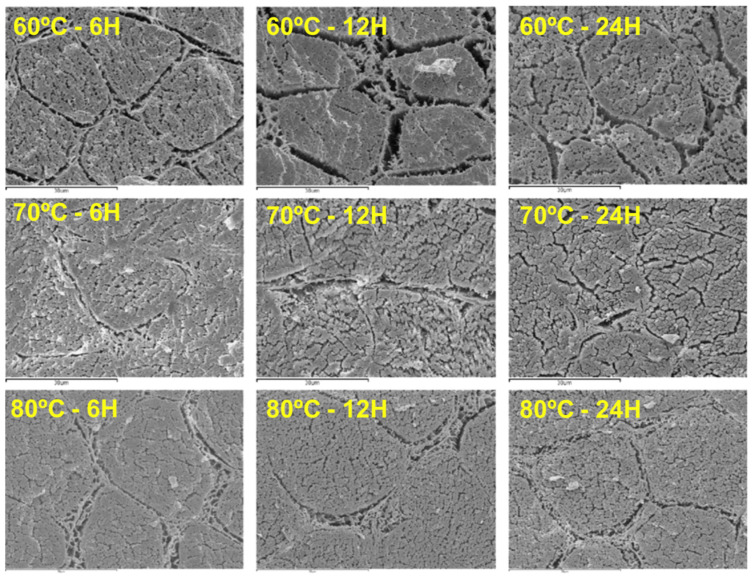
Microstructure of sous-vide cooked Longissimus Dorsi muscle of lambs at three different temperature-time combinations (2000× magnification) [82].

In addition to the above methods, scanning electron microscopy is another technique for visually demonstrating changes in muscle fibres, thereby reflecting textural changes. Figure 2 shows the microstructure of the lamb Longissimus Dorsi muscle cooked under different time-temperature combinations. At 60 °C, gaps between myofibres were visible, and granular deposits in the spaces between myofibres were prominent. The perimysium and endomysium became granular at this temperature. Wattanachant, Benjakul, and Ledward [101] attributed this shrinkage to the thermal denaturation of intramuscular collagen, occurring within the temperature range of 60.7–61.7 °C. Further heating resulted in the formation of a denatured collagen gel around each myofibre, which helped maintain cell shape despite water loss. At 70 °C, the meat structure became denser with more compact fibre arrangements, and the granular deposits became less distinct. The connective tissue is denatured, with subsequent formations filling the spaces between myofibres, endomysium, and fibre bundles. This intense granulation could be due to sarcolemma distortion [102]. Under 80 °C, gaps between myofibres were evident, and the spaces between the endomysium and myofibres were filled with granular deposits. The perimysium and endomysium gelatinised at this temperature. Compared to 60 °C, samples cooked at 80 °C exhibited a swollen volume, with a specific reduction in myofibre diameters.

The degree of myoglobin denaturation is related to heating temperature. Higher cooking temperatures result in a higher denaturation ratio, which, in turn, leads to lower a* values. Although the cooking temperature for Grilling (GR) steaks was the highest, their myoglobin denaturation ratio was lower than that of Boiling cooking (BO) steaks. This might be because heating was stopped when the central temperature of the GR steaks reached just 72 °C [103].

#### 3.2.3. Changes in Some Specific Proteins

Under sous-vide cooking temperatures, some endogenous enzymes remain bioactive, while others are rapidly inactivated. For example, m-calpain and μ-calpain are inactivated after 10 min of incubation at 55 °C. Cathepsins B and L are key endopeptidases that catalyse the hydrolysis of internal peptide bonds in proteins, and they are located within the lysosomes of muscle cells. When cooking below 63 °C, the residual activity of cathepsins B and L is positively correlated with temperature and negatively correlated with heating time. These enzymes can also be detected in cooking losses, indicating their release from lysosomes. It has also been demonstrated that the proteolytic activity in beef cooked at 60 °C is higher than that in beef cooked at 50 °C, suggesting that increased activity of certain endogenous enzymes can influence the texture of sous-vide cooked meat. The myofibrillar fragmentation index is an indicator of myofibrillar protein degradation in the I band of the sarcomere. A higher myofibrillar fragmentation index is associated with increased meat tenderness and reduced shear force [104]. The weakening of myofibrillar and connective tissue proteins contributes to enhanced collagen solubility and tenderness in the M. biceps femoris. The amount of soluble collagen is positively correlated with meat tenderness [105].

For beef, one-third of the proteins in muscle consist of α-actinin, tropomyosin, and troponin [106]. Myosin may be further broken down by proteolytic enzymes that are more active at sous-vide temperatures [107]. Russian sturgeon flesh is highly perishable due to microbial spoilage. Results on Russian sturgeon flesh showed that after sous-vide cooking at 60 °C, protein oxidation and degradation were aggravated, with reductions in myosin light chain, myosin heavy chain, and sulfhydryl content. Proteomic analysis indicated that differentially expressed proteins were primarily involved in cellular components and biological processes. Myofibrillar proteins, as the main functional components accounting for >65% of total protein in muscle, play a vital role in meat processing. Myosin is sensitive to oxidative attack due to its several cysteine residues. These results suggest that sous-vide cooking pretreatment may promote protein oxidation and degradation, potentially caused by the formation of free radicals.

#### 3.2.4. Changes in Amino Acids During Sous-Vide Cooking

Amino acids can degrade during thermal processing. Certain amino acids, such as lysine, cysteine, and glutathione, can also react with carbonyl compounds. Compared with traditional high-temperature cooking methods, sous-vide cooking significantly inhibits amino acid oxidation and leaching loss through juices, owing to its low temperature and minimal oxygen exposure. Furthermore, most enzymes lose their activity at temperatures above 40 °C, become irreversibly inactivated between 60 °C and 70 °C, and remain irreversibly inactivated at temperatures exceeding 70 °C. Mild and sustained thermal stimulation activates endopeptidases such as cathepsin B/L and calpain-2, inducing controlled hydrolysis of myofibrillar and collagen proteins [67]. This process increases the total content of free amino acids, with alanine, glycine, and lysine showing the most significant elevations, thereby enhancing tenderness, water-holding capacity, and digestibility [29]. In the study of Bhat et al. [29], the content of nearly all amino acids increased in the samples subjected to sous-vide cooking. Liu et al. [108] also demonstrated that sous-vide cooking retained higher levels of free amino acids, thereby facilitating the formation of more umami and sweet-tasting amino acids compared with traditional high-temperature cooking.

It has been shown that higher temperature-time combinations promote the formation of volatile compounds from these reactions [81]. Nyam et al. [109] demonstrated that the diffusion coefficients of free amino acids during the sous-vide cooking of chicken are smaller than those in the conventional cooking. This implies that the release of amino acids is slower during sous-vide cooking, contributing to the better taste of the chicken.

### 3.3. Water Changes During Sous-Vide Cooking

Water constitutes the largest proportion of meat, encompassing free water, capillary water, and bound water. Bound water exists in food and forms bonds with non-aqueous components via hydrogen bonding, and it is the most tightly bound water to non-aqueous components in food. Free water refers to water that has little or essentially no interaction with non-aqueous ingredients in food. Capillary water is the water present in the interstitial spaces of organisms. During thermal processing, the secondary structure of proteins undergoes denaturation, hydrogen bonds are disrupted, and the restraint on water molecules is weakened. This leads to water loss, which in turn affects the texture of the food.

Juiciness is a crucial parameter for consumers. Compared to traditional thermal cooking, sous-vide-cooked meat exhibits higher juiciness, owing to minimal dehydration at lower cooking temperatures. In comparison with traditionally cooked beef, sous-vide cooked beef has a paler, moister surface, a coloured cut surface, and a juicier texture [110]. Turner and Larick [111] reported that sous-vide cooked chicken at 77 °C was significantly juicier and had a more pronounced soapy/bitter taste than chicken cooked to 94 °C. It is suggested that the juiciness of chicken can be enhanced through sous-vide cooking. Water loss during thermal processing can be primarily attributed to two processes, namely osmosis and the reduction in the water-holding capacity of proteins. Since sous-vide cooking retains more moisture in food than traditional cooking, the protein/lipid ratio is lower in traditionally cooked meat, which results from the variations in weight loss. Jeong et al. [34] noted that sous-vide cooking at 71 °C resulted in a higher moisture content and lower cooking loss compared to 61 °C, primarily due to the shrinkage of perimysium and myofibrils at higher temperatures. During thermal processing, the denaturation and shrinkage of certain structure-related proteins can lead to cooking loss. Water loss caused by the shrinkage of myofibres and connective tissue occurs at approximately 53 °C and 63 °C, respectively. Within the temperature range of 60–80 °C, there is a correlation between temperature and weight loss. Roldán et al. [77] observed that lamb loins cooked at 60 °C and 70 °C had a higher moisture content than those cooked at 80 °C. Sánchez del Pulgar et al. [63] found that during sous-vide cooking, the weight loss increased as the temperature increased. In the range of 40–90 °C, myofibrillar proteins denature and shrink with increasing temperature, while collagen shrinks between 56 °C and 62 °C [91]. Up to 60 °C, myofibres shrink transversely, widening the gaps between them. As the temperature continues to rise, the myofibres shrink longitudinally, leading to significant water loss, with the degree of this contraction increasing alongside temperature.

### 3.4. Other Changes During Sous-Vide Cooking

The diffusion coefficients of organic acids, sodium chloride, and nucleic acids have been shown to be lower during sous-vide cooking than those in traditional thermal cooking. The slower release of these flavour compounds contributes to a better taste in meat. Sut et al. [112] assessed the effects of four different cooking methods on porcine Longissimus lumborum, and found that the sous-vide method retained the highest levels of vitamin B in the meat. The thiamin content of sous-vide-cooked chicken breast was preserved at 98%. Sous-vide-cooked turkey breast also exhibited higher vitamin retention [113]. Sous-vide cooking of meat has grown in popularity in recent years due to its positive impact on tenderness and juiciness. In addition to the aforementioned sensory properties, meat cooked at temperatures up to 70 °C could also maintain higher concentrations of minerals compared to convection methods such as broiling [114].

## 4. Future Prospects

Over the past few decades, research into sous-vide cooking has attracted growing attention. The changes in certain types of meat during sous-vide cooking have been explored at a sensory level, and there have also been studies into the mechanisms behind changes in food components, including proteins, lipids, water, and other constituents. Comparisons between traditional cooking methods and sous-vide cooking have likewise been investigated. However, several areas warrant further exploration:

• While meats primarily consist of similar components (water, protein, and lipid), the specific characteristics of different meats can vary. To date, most studies have focused on the selection and optimization of sous-vide cooking for a single type of meat. Thus, the effects of sous-vide cooking across different species should be studied.
• There are existing standards for evaluating ready-to-eat food, with most emphasizing microbial aspects. However, as demands for high food quality continue to rise, grading standards for sous-vide-cooked meat require further discussion and definition. This should include the development of quantitative methods and a scientific evaluation system to assess the quality of such products.
• Research has explored the molecular mechanisms underlying quality changes in sous-vide cooked meat, particularly regarding flavour development. Studies have examined changes in certain lipids and proteins, with previous work discussing and quantifying specific lipid oxidation products and protein denaturation. Nonetheless, a more systematic and in-depth exploration is needed. Using omics approaches and precise instrumental analysis, components of sous-vide-cooked meats could be evaluated with high resolution. For example, mass spectrometry-based proteomics can provide detailed qualitative and quantitative analyses of individual proteins, clarify the relationships between texture changes and specific structural proteins, and enhance the understanding of how sous-vide cooking maintains meat texture. Investigating the changes in the activity and metabolic pathways of endogenous enzymes during sous-vide cooking can also shed light on biological activities. Advanced techniques such as low-field nuclear magnetic resonance and nuclear magnetic resonance imaging can elaborate on the moisture migration patterns, aiding comprehension of water retention mechanisms. These methods would enable more precise explanations of molecular-level changes during sous-vide cooking.
• Most previous studies have focused on sous-vide cooking’s effects on pure meat, yet food processing often involves combinations of multiple ingredients rather than simple meat cuts. Thus, attention should be paid to the impact of additives and interactions between different raw materials. For instance, how salt ions affect meat’s water retention (whether positively or negatively), how to preserve or counteract this effect, and how the Maillard reaction proceeds in the presence of sugar and its impact on meat texture and flavour.
• Previous research has confirmed that time and temperature are critical parameters influencing the quality of sous-vide cooked meat, with certain biochemical reactions (e.g., protein denaturation and enzyme inactivation) being irreversible and dependent on strict temperature conditions. This places higher demands on the temperature control capabilities of cooking equipment. Research into instruments with superior temperature control can facilitate the production of sous-vide foods with more consistent and controllable quality.

## 5. Conclusions

High-quality products produced using sous-vide cooking meet the growing demand for minimal cooking. While both time and temperature are key factors, temperature often plays a more critical role in determining the final quality of cooked meat. As a complex food system, each component of meat undergoes distinct biochemical reactions under various temperature-time combinations. These components also interact with one another, as do the further reactions between primary reaction products. The outcomes of these complex reactions collectively shape the sensory properties of sous-vide-cooked products. Partial protein denaturation endows meat with textures that differ from those achieved through traditional thermal cooking. Controlling the temperatures at which collagen aggregates, myofibrils contract, or certain endogenous enzymes are inactivated can significantly influence the texture and moisture migration. Products of protein oxidation and lipid oxidation will also react individually or with specific amino acid groups to generate unique flavours. Additionally, the combined effects of moisture and protein contribute to the colour variations observed in meat products cooked under different temperature-time combinations.

Although sous-vide cooking has seen increasing application in both industrial settings and restaurants, further research should focus on more precise component separation and identification, the establishment of unified standards, and improvements in the accuracy of related instruments and equipment.

## Figures and Tables

**Table 1:** Recent research on sous-vide cooking parameters for meat and seafood.

Subject	Temperature	Time	Reference
Yellowfin tuna	55, 60, 65 °C	30 min	Yuan et al., 2025 [15]
Pork loin	50, 55, 60 °C	12, 24 h	Hwang et al., 2019 [16]
Beef	55 °C	45 min	Sun et al., 2019 [9]
Beef	55 °C	2, 10, 30 h	Trbovich et al., 2017 [17]
Beef	55, 70 °C	2, 8 h	Trbovich et al., 2017 [18]
Goose meat	60, 80 °C	4, 6, 12 h	Wereńska, 2024 [6]
Chicken breast	55, 57.5, 60 °C	1 h	Karyotis et al., 2017 [19]
Döner kebabs	57.5, 60, 62.5, 65 °C	1 h	Haskaraca et al., 2019 [20]
Pork	58 °C	60, 90 min	Kehlet et al., 2017 [21]
Beef briskets	60 °C	12, 18, 24 h	Alahakoon et al., 2019 [22]
Atlantic mackerel	60, 75, 90 °C	10, 15, 20 min	Cropotova et al., 2019 [23]
Spent buffalo meat	55, 65, 95 °C	480, 300, 45 min	Haq et al., 2024 [24]
Beef	39, 49, 59, 70 °C	1, 4 h	Uttaro et al., 2019 [25]
Beef brisket	60, 65, 70 °C	24, 48, 72 h	Alahakoon et al., 2018 [26]
Lamb and goat	60 °C	6, 8, 10, 12 h	Gawat et al., 2024 [27]
Captive pirarucu	60 °C	9.48 min	Hernández et al., 2017 [28]
Dairy cows	60 °C	4.5, 10 h	Bhat et al., 2020 [29]
Beef	60 °C	24 h	Chian et al., 2021 [30]
Beef	60 °C	4 h	Modzelewska-Kapituła et al., 2019 [31]
Goat	60, 65, 70 °C	6, 12 h	Ismail et al., 2019 [32]
Pirarucu	60 °C	9.48 min	Hernández et al., 2020 [33]
Pork ham	61 °C	45 min	Jeong et al., 2018 [34]
Pork ham	61, 71 °C	45, 90 min	Jeong et al., 2018 [35]
Beef shank cuts	55, 65, 75 °C	2, 5, 8, 12, 24 h	Gámbaro et al., 2023 [36]
Tambaqui	65 °C	12.5 min	Ramos et al., 2017 [37]
Bovine live	65 °C	2 h	Da Silva et al., 2017 [38]
Trout fillets	65, 75, 85 °C	60, 75, 90, 120, 135, 150 min	Oz, F., & Seyyar, E., 2016 [39]
Seerfish	70, 80, 90 °C	5, 10, 15 min	Singh et al., 2016 [40]
Beef brisket	70 °C	30 min	Zhu et al., 2018 [41]
Pork loin	70 °C	1, 2, 4, 6, 8 h	Perez-Palacios et al., 2019 [42]
Atlantic mackerel	70, 80 °C	10, 20 min	Cropotova et al., 2019 [43]
European sea bass	85 °C	20 min	Nieva-Echevarría et al., 2017 [44]

**Table 2:** The effect of sous-vide cooking meat/seafood on lipid oxidation.

Material	Temperature	Time	Effect
Nile Tilapia	50, 60 °C	30, 45, 60 min	Compared with traditional steaming methods, lipid oxidation is lower, and lipid oxidation increases with increasing cooking temperature or processing time [52].
Russian sturgeon	40, 50, 60 °C	10 min	Treatment at 60 °C had an adverse effect on lipid oxidation in Russian sturgeon meat, resulting in increased peroxide values and accelerated changes in conjugated diene values, indicating a high degree of oxidation [84].
Atlantic mackerel	70, 80 °C	10, 20 min	Higher temperatures and cooking times typically accelerate lipid oxidation [43].
Lamb meat	75 °C	35 min	Compared with traditional barbecuing, vacuum low-temperature cooking prevents TBARS and oxysterols compared to grilled patties, sous-vide cooking inhibited (*p* < 0.05) the formation of malondialdehyde, and 7α- and 7β-hydroxycholesterol, and lowered the cholesterol oxidation ratio during heated display [58].
Pork hams	55, 58, 60 °C	3, 5, 8 h	Sous-vide cooking method at 58 °C for 5 h reduces lipid peroxidation [85].
Reduced-salt chicken breast ham	60 °C	2 h	Compared with traditional methods, sous-vide cooking can delay lipid oxidation [86].

## Data Availability

No new data were created or analyzed in this study. Data sharing is not applicable to this article.
